# Fusaoctaxin A, an Example of a Two-Step Mechanism for Non-Ribosomal Peptide Assembly and Maturation in Fungi

**DOI:** 10.3390/toxins11050277

**Published:** 2019-05-16

**Authors:** Klaus Ringsborg Westphal, Katrine Amalie Hamborg Nielsen, Rasmus Dam Wollenberg, Mathias Bonde Møllehøj, Simone Bachleitner, Lena Studt, Erik Lysøe, Henriette Giese, Reinhard Wimmer, Jens Laurids Sørensen, Teis Esben Sondergaard

**Affiliations:** 1 Department of Chemistry and Bioscience, Aalborg University, 9220 Aalborg, Denmark; kw@bio.aau.dk (K.R.W.); kah@bio.aau.dk (K.A.H.N.); rwo@bio.aau.dk (R.D.W.); mathiasbm@hotmail.com (M.B.M.); hgiese@bio.aau.dk (H.G.); rw@bio.aau.dk (R.W.); 2 Department of Applied Genetics and Cell Biology, University of Natural Resources and Life Sciences, Vienna (BOKU), Konrad-Lorenz-Straße 24, Tulln an der Donau 3430, Austria; simone.bachleitner@boku.ac.at (S.B.); lena.studt@boku.ac.at (L.S.); 3 Division of Biotechnology and Plant Health, Norwegian Institute of Bioeconomy Research, 1433 Ås, Norway; erik.lysoe@nibio.no; 4 Department of Chemistry and Bioscience, Aalborg University, Niels Bohrs Vej 8, 6700 Esbjerg, Denmark; jls@bio.aau.dk

**Keywords:** Fusarium, NRPS, secondary metabolites, Mycotoxins, ABC-transporter, peptidase

## Abstract

Fungal non-ribosomal peptide synthetase (*NRPS)* clusters are spread across the chromosomes, where several modifying enzyme-encoding genes typically flank one *NRPS*. However, a recent study showed that the octapeptide fusaoctaxin A is tandemly synthesized by two NRPSs in *Fusarium graminearum*. Here, we illuminate parts of the biosynthetic route of fusaoctaxin A, which is cleaved into the tripeptide fusatrixin A and the pentapeptide fusapentaxin A during transport by a cluster-specific ABC transporter with peptidase activity. Further, we deleted the histone H3K27 methyltransferase *kmt6*, which induced the production of fusaoctaxin A.

## 1. Introduction

**Figure 1:**
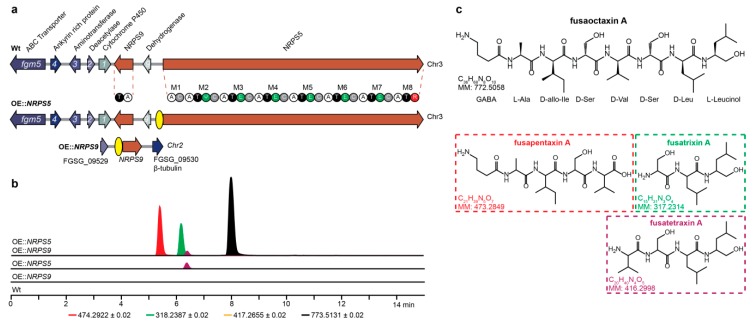
(**a**) Organization of the fusaoctaxin A gene cluster (*fgm1-5 + 9*) in *F. graminearum* and activation of key enzymes by in situ overexpression of *NRPS5* and ex situ overexpression of *NRPS9*. The yellow ovals show positions of inserted constitutive promoters. (**b**) Extracted ion chromatograms of the protonated ions [M+H]^+^ for fusapentaxin A (red), fusatrixin A (green), fusatetraxin A (purple) and fusaoctaxin A (black) in *F. graminearum* wild type and overexpression strains. (**c**) Structures of the different fusaoctaxin A’s with chemical formula and monoisotopic mass (MM).

Filamentous fungi present a rich source of non-ribosomal peptides (NRPs) that are used in the battle for survival in their natural habitats and they have a huge impact on human health: penicillin and cyclosporine are examples of NRPs [1,2]. The vast majority of NRPs is synthesized by a single multi-modular non-ribosomal peptide synthetase (NRPS) that is in concert with tailoring enzymes. A minimal NRPS module consists of an adenylation domain (A) for the recognition and activation of a specific amino acid substrate, which is transferred by a peptidyl carrier protein domain (T or PCP) to the condensation domain (C) where a peptide bond is formed between the activated amino acid and the preceding elongated chain [3]. Additionally, auxiliary domains may also be present in a module, such as the epimerization domain (E), which inverts the stereo chemistry of the peptide alpha carbon, thus changing the l-configuration to the d-configuration. The final module typically contains a thioesterase domain (TE) or a reductase domain [4]. The TE domain facilitates product release through cyclization or hydrolysis, and the R domain utilizes NAD(P)H for reductive product release, which results in a carboxylic acid, an aldehyde, or/and alcohol [5]. *NRPSs* are tightly regulated and, consequently, the majority are not expressed during standard growth conditions, not in the laboratory and few during plant infection [6,7]. Recently, two non-ribosomal virulence factors have been identified in the omnipresent plant pathogen *Fusarium graminearum;* Gramillin was identified and it showed to be encoded by *NRPS8* [8] and the *NRPS5*/*NRPS9* gene cluster was shown to code for the linear octapeptide fusaoctaxin A [9]. Comparative analyzes of *NRPSs* in the *Fusarium* genus have previously shown that *NRPS5* and *NRPS9* are present in several species including *F. avenaceum*, *F. acuminatum*, *F. culmorum*, *F. pseudograminearum,* and in one of twelve *F. oxysporum* strains (FOSC 3-a) [10,11]. Synteny mapping shows that the two genes reside in an eight-member gene cluster, which is highly conserved (Figure S1). In addition to *NRPS5* and *NRPS9*, the gene cluster comprises genes that are predicted to encode a dehydrogenase *(fgm9)*, a cytochrome P450 monooxygenase *(fgm1)*, a deacetylase *(fgm2)*, an amino transferase *(fgm3)*, a transcription factor with ankyrin-rich repeats *(fgm4),* and an ABC transporter *(fgm5)*. Jia LJ et al. epitopically overexpressed the cluster specific transcription factor *fgm4* in order to express and characterize fusaoctaxin A [9]. Additionally, they identified fusaoctaxin A as an important virulence factor that is required for cell-to-cell invasiveness. During the infection of crops and cereals, *F. graminearum* produces several mycotoxins, which can have serious impact on human and animal health. The aim of this study was to investigate possible post NRPS biosynthesis processes for fusaoctaxin A. To accommodate this, we overexpressed the two *NRPSs* in individual mutants by introducing a constitutive promoter upstream of the *NRPS5* gene encoding the eight module NRPS5 and by expressing the *NRPS9* gene encoding the non-module two domain (A and T) NRPS9 in a position that is close to the housekeeping gene, β-tubulin (Figure 1a). The mutations were additionally introduced into the same mutant, thus resulting in a double mutant overexpressing both NRPSs.

## 2. Results and Discussion

### 2.1. Generation of Overexpression Stains

The transformation vectors were constructed by USER friendly cloning [12], and second generation Illumina and third generation MinION Nanopore sequencing verified that the resulting mutants were correct (Figure S2). The secondary metabolite extracts from three overexpression strains OE::*NRPS5*, OE::*NRPS9,* and OE::*NRPS5*::*NRPS9* were analyzed by HPLC-HRMS and compared to the wild type (Wt) (Figure 1b). The chromatogram of the OE::*NRPS5*::*NRPS9* extract contained several intense peaks eluting between five and nine minutes, none of which could be detected in the chromatograms of the Wt or OE::*NRPS9* extracts. Three peaks: fusaoctaxin A ([M + H]^+^ 773.5131 Da, C_36_H_68_N_8_O_10_), fusapentaxin ([M + H]^+^ 474.2922 Da, C_21_H_39_N_5_O_7_), and fusatrixin ([M + H]^+^ 318.2387 Da, C_15_H_31_N_3_O_4_) were exclusively observed in the *OE::NRPS5::NRPS9* metabolite extract. Notably, fusatetraxin ([M + H]^+^ 417.2655 Da, C_20_H_40_N_4_O_5_) was observed in the OE::*NRPS5,* as well as in the *OE::NRPS5::NRPS9* metabolite extracts (Figure 1c; Figure S3a,b).

Fusaoctaxin A was isolated from *OE::NRPS5::NRPS9* and the structure was confirmed by one-dimensional (1D) and two-dimensional (2D) NMR spectroscopy and by Marfey’s method (Figures S4–S6) to be GABA-l-Ala-d-*allo*-Ile-d-Ser-d-Val-d-Ser-d-Leu-l-Leucinol, as found by Jia LJ and co-workers [9].

### 2.2. Structure Elucidation

The arrangement of l- and d- residues in fusaoctaxin A correlates with epimerase domains (E) in NRPS5, except for the second module that is supposedly responsible for the incorporation of alanine. However, the E domain of the second module is truncated when compared to other functionally active E domains that explain the l-configuration observed. The molecular formulae of fusapentaxin A and fusatrixin A equaled that of fusaoctaxin A, with the addition of water suggesting hydrolytic cleavage of fusaoctaxin A. These structures were examined by MS/MS, from which the amino acid sequences could be assigned from a-, b-, and y- ions. Assignment of the bbCID spectrum of fusapentaxin A gave the amino acid sequence GABA-Ala-Ile-Ser-Val (Figure S7a,d). A delta-mass of 113.0843 Da, as observed in the bbCID spectrum of fusapentaxin A, could be from either leucine or isoleucine, however, when comparing with fusaoctaxin A, it suggests isoleucine as the third residue. Following the same procedure, the residue sequence for fusatrixin A and fusatetraxin A were solved as Ser-Leu-Leucinol and Val-Ser-Leu-Leucinol, respectively (Figure S7b–d).

### 2.3. Regulation by H3K27 Methylation

The deletion of the *kmt6* gene (FGSG_15795) in *F. graminearum* eliminates H3K27 trimethylation and thereby induces the expression of several otherwise silent secondary metabolite gene clusters [13]. We used targeted gene replacement to disrupt the *kmt6* gene to test whether H3K27 methylation is responsible for regulation of the fusaoctaxin A gene cluster. This led to significantly elevated levels of fusapentaxin A and fusatrixin A when compared to the Wt. This suggests that the fusaoctaxin A gene cluster is silenced by H3K27 trimethylation in the Wt and it is in accordance with earlier observations that *kmt6* affects the expression of *NRPS9* [13].

### 2.4. Clevage by Peptidase

**Figure 2:**
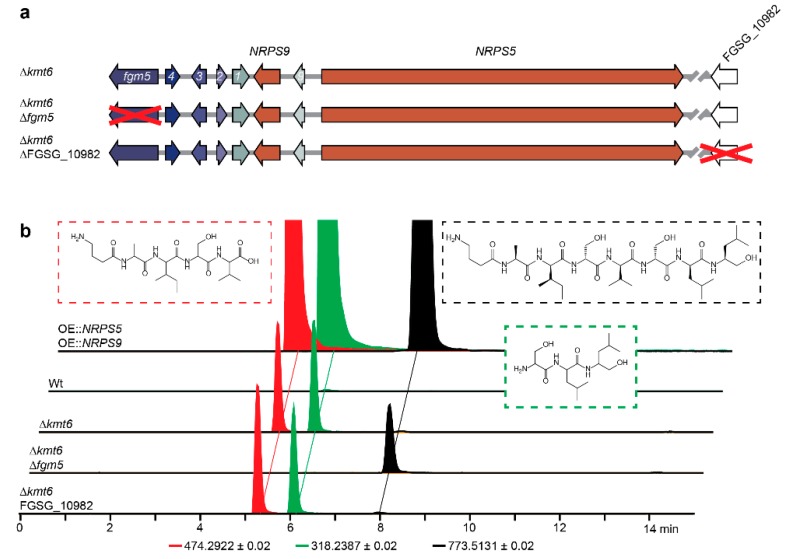
(**a**) Schematic illustration of the fusaoctaxin A gene cluster and deletion of the ABC transporter (*fgr5*) and a putative peptidase (FGSG_10982) located near the cluster in a *kmt6* deleted *F. graminearum* strain. (**b**) Extracted ion chromatograms of the protonated ions [M+H]^+^ for fusapentaxin A (red), fusatrixin A (green) and fusaoctaxin A (black).

The *kmt6* deletion mutant was further used to identify the origin of fusapentaxin A and fusatrixin A. Bioinformatic analysis of the gene cluster and neighboring genes gave two possible candidates for gene deletion. A putative peptidase (FGSG_10982) found seven genes downstream of *NRPS5*, and an ABC transporter (*fgm5*), which has the homology to a SunT domain described as a bacteriocin/lantibiotic exporter with peptidase activity (Figure 2a). The deletion of FGSG_10982 in the Δ*kmt6* background did not influence the production of fusapentaxin A and fusatrixin A. Deletion of *fgm5* resulted in a substantial increase of fusaoctaxin A, while the production of fusapentaxin A and fusatrixin A was no longer detectable (Figure 2b).

### 2.5. Virulence and Bioactivity

**Figure 3:**
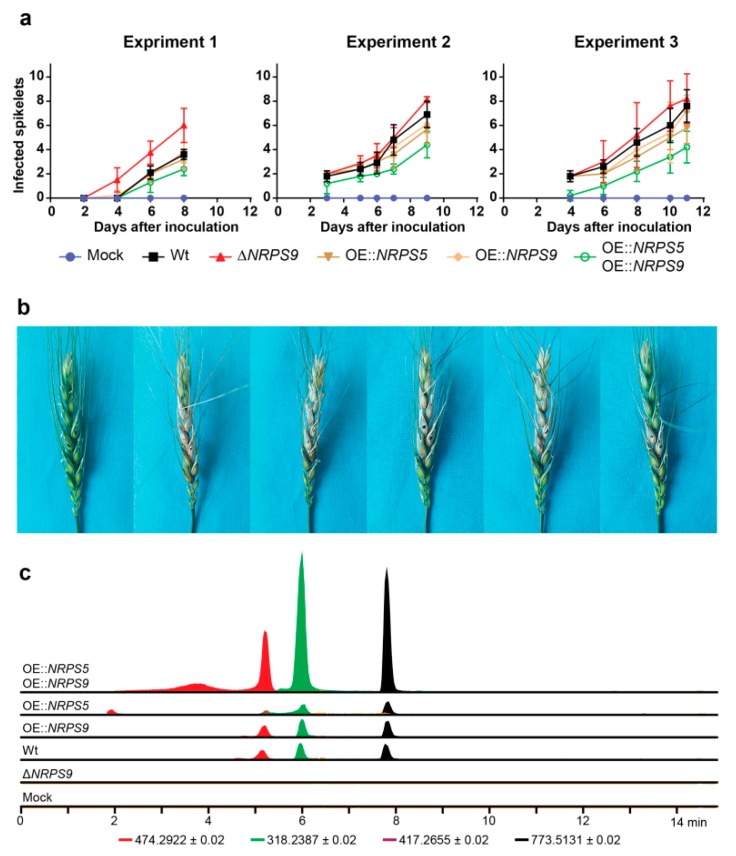
(**a**) Wheat head infection of *F. graminearum* strains in three independent experiments. Each point represent means of five heads and error bars are based on standard deviation. (**b**) Representative wheat heads collected at harvest in experiment 2. (**c**) Extracted ion chromatograms of metabolite extracts of wheat heads from experiment 2 for fusapentaxin A (red), fusatetraxin A (yellow), fusatrixin A (green), and fusaoctaxin A (black).

Fusaoctaxin A may play a role in plant pathogenicity expressed in *F. graminearum* during growth inside developing wheat coleoptiles [14]. Further support for this hypothesis is that the deletion of *fgm5* significantly reduces the virulence of *F. graminearum* on wheat, barley, and maize heads [15]. Additionally, transcriptome data from 176 microarray samples from 55 various experiments (Figure S8a,b) show that the gene cluster is active during the infection of wheat and barley. The role of fusaoctaxin A peptides on virulence in wheat was examined by the inoculation of wheat heads with the *NRPS9* deletion mutant and the three different overexpressing mutants (Figure 3a,b). The most virulent strain was Δ*NRPS9*, followed by the Wt, OE::*NRPS5*, and OE::*NRPS9*. The combined overexpression of *NRPS5* and *NRPS9* resulted in reduced virulence as compared to the Wt in both experiments (*p* < 0.05). Chemical analyzes failed to detect fusaoctaxin A’s in the Δ*NRPS9* strain suggesting that the fusaoctaxin A’s are not important virulence factors (Figure 3c). It is plausible that an overproduction of a metabolite that aids in infection does not necessarily result in a hypervirulent strain. Thus, we do not see an effect in the OE strains, while the hypovirulence might very well be present in a delta strain. Our results are not completely in line with recently published data [9], as fusaoctaxin A has been claimed to be an important virulence factor. The diverse results could be due to different overexpression mutants and a different variety of wheats. Fusaoctaxin A and the cleaved products: fusapentaxin A and fusatrixin A were present in high levels in *OE::NRPS5* and *OE::NRPS9* and at comparably lower levels in the Wt and single overexpression strains. An explanation for the reduced virulence could be a toxic effect of fusaoctaxin A, the tri-peptide, or the penta-peptide. To test this hypothesis, we chemically synthesized fusaoctaxin A, fusapentaxin A, and fusatrixin A, and tested their toxicity against selected *Fusarium* species using real time microscopy (Figure S9a–h). We could not detect a toxic effect against a fusaoctaxin A producer (*F. graminearum*) or a non-producer (*F. solani*), making the bioactivity of fusaoctaxin A and the cleaved products a question for the future.

### 2.6. Model for Maturation

Based on our results, we confirm the biosynthetic route for fusaoctaxin A, where two NRPSs work tandemly to synthesize fusaoctaxin A. Jia LJ et al. suggest that NRPS9 delivers the first GABA, and thereby initiates the synthesis [9]. The first module in NRPS5 only contains a single truncated A domain when compared to the other adenylation domains and might not be fully functional. In fungi, only the ergopeptine synthesis with two NRPSs encoded from the same cluster has been described. Ergopeptine synthesis is initiated by a stand-alone NRPS that grabs, modifies, and delivers the first amino acid to a three module NRPS that result in a final tri-peptide [16,17]. This is more common in bacteria, where several NRPSs from the same operon have been shown to produce common peptides [18,19].

The full-length peptide fusaoctaxin A consists of eight amino acids and it is the only variant that is detected when a cluster-specific ABC transporter is deleted. The overexpression of NRPS5 alone produces fusatetraxin A, which suggests that the A domain in module 5 can incorporate valine and initiates the synthesis of the last part of the peptide. This observation would be in line with the latest structural observation that reveals high mobility and rotation within NRPS enzymes [20], and it could explain why we observe the tetra-peptide in the OE::*NRPS5*. Secondly, the octa-peptide fusaoctaxin A is cleaved to a penta- and a tri-peptide by a cluster specific ABC transporter with peptidase activity. This activation or removal of signal peptides is common in ribosomal peptides and proteins [21]. It has been observed in few bacterial cases, where the NRPs are matured by proteolytic cleavage by d-asparagine specific carboxy-peptidases during transport across membranes [22,23]. If the final peptide is toxic, then this mechanism provides an evolutionary advantage to the host [24]. In line with this, some plants produce secondary metabolites that inhibit ABC transporters as part of a defense mechanism against fungal attacks, which would provide a status quo in plant fungal interactions [25]. The enigma of how hosts cope with toxic intracellular compounds may, in part, be answered by this transport cleavage and it would not require the involvement of intracellular vesicles, such as aflatoxisomes [26].

It is problematic to predict the primary NRPs structure based on the module structure of the NRPS, but, according to the collinearity rule, the number of amino acids reflects the number of modules in the NRPS. There are several exceptions, particularly in iterative *NRPSs* that assemble siderophores. Jia et al. found that *NRPS5* and *NRPS9* followed the collinearity rule and produced an eight amino acid long peptide fusaoctaxin A by the overexpression of a cluster specific transcription factor [9]. We found and identified the same compound fusaoctaxin A, but we did also observe that the compound was cleaved into a penta- and a tri-peptide, and that the cleavage could be inhibited by knocking out the cluster specific ABC transporter harboring peptidase activity.

## 3. Conclusions

In summary, we have shown that H3K27 methylation controls the *fusaoctaxin A* gene cluster and that the octapeptide fusaoctaxin A is cleaved by a cluster specific ABC transporter to a pentapeptide fusapentaxin A and a tripeptide fusatrixin A. Whether this cleavage is part of protection from own toxins or matureness of virulence factors are important questions for the future.

## 4. Materials and Methods

### 4.1. Cells and Chemicals

General cloning steps and vector amplification was carried out while using chemically competent *Escherichia coli* XL1 Blue genotype: recA1 endA1 gyrA96 thi-1 hsdR17 supE44 relA1 lac [F’ proAB lacI^q^Z∆M15 Tn10 (Tet^r^)] obtained from Stratagene (Agilent, Glostrup, Denmark). Uracil-specific excision reagent (USER) friendly cloning was carried out using chemically competent *E. coli* JM109 genotype: e14^–^ (McrA^–^) recA1 endA1 gyrA96 thi-1 hsdR17 (r_K_^–^ m_K_^+^) supE44 relA1 ∆(lac-proAB) [F´ traD36 proAB lacI^q^Z∆M15] obtained from Stratagene. *Agrobacterium tumefaciens* mediated transformation (ATMT) was carried out using ElectroMAX^TM^ *A. tumefaciens* LBA4404 cells that were obtained from Invitrogen, Thermo Fisher Scientific Inc. catalog number: 18313-015, Hvidovre, Denmark. F. graminearum PH-1 (NRRL31084) was obtained from the Agricultural Research Service Culture Collection, National Center for Agricultural Utilisation Research. All of the primers were obtained from MWG Biotech, (Eurofins, Ebersberg, Germany) (Table S1). All the plasmid sequencing was carried out at MWG Biotech, Eurofins. All PCR products were purified using QIAquick PCR purification Kit (Qiagen, Hilden, Germany) or Gel Extraction Kit (Qiagen). Plasmids purification was carried out using QIAprep Spin Miniprep Kit (Qiagen). Fungal DNA for PCR reactions were extracted using DNeasy Plant Mini Kit (Qiagen). All of the DNA concentrations were estimated using NanoDrop-1000 (Thermo Scientific, Hvidovre, Denmark).

### 4.2. Cluster Prediction

The *F. graminearum* chromosome III (accession number: HG970334.1) into Antismash 3.0 Fungal Version [27], where the fusaoctaxin A gene cluster was identified using the optional Cluster-border prediction based on transcription factor binding sites (CASSIS) settings [28]. The genomic regions of other *Fusarium* species containing the gene cluster were imported into CLC Main Workbench 7 (Qiagen, Vedbæk, Denmark) and used to generate a Synteny plot.

### 4.3. Transcriptional Analysis

Hierarchical clustering of the fusaoctaxin A gene cluster and neighbouring genes was performed in CLC Genomics Workbench 6 using 175 microarray samples from 55 experiments, as previously described [6,29].

### 4.4. Cloning

The plasmid for kmt6 deletion was generated using yeast recombinational cloning [30]. For this, the upstream and downstream sequences of kmt6 were amplified from *F. graminearum* PH-1 genomic DNA with the primer pairs FGSG_15795_5F//FGSG_15795_5R and FGSG_15795_3F//FGSG_15795_3R, respectively. The hygromycin B resistance cassette was amplified while using the primer pair hph-F and hph-R from pCSN44. The Saccharomyces cerevisiae strain FY834 [31] was transformed with the obtained fragments, as well as with the EcoRI/XhoI-restricted pRS426 [32] yielding p∆kmt6. For genome editing, protoplasts were prepared from the wild-type strain PH-1. Transformation was carried out, as previously described [33]. The construction of vectors for over-expression and knockout of selective NRPS genes was performed using the well-established USER friendly cloning technique [12]. This method relies on a single four fragment cloning step to integrate the two sequences into a recipient vector, followed by in vivo ligation. ATMT was used for fungal genome editing through homologous recombination facilitated by homologous regions, as described by Frandsen et al. [12]. The OE::*NRPS5::NRPS9* mutant was constructed using a U-GOTL template vector [34] or the insertion of the 2514 bp NRPS9 gene into the tubulin locus of an existing OE::*NRPS5* mutant. The *NRPS9* gene was amplified using *NRPS9* (tubulin) forward and reverse primers (Table S1). The U-GOTL::NRPS9 plasmid was introduced to spores from the OE::*NRPS5* strain by ATMT. Δfgm5Δkmt6 and ΔFGSG_10982Δkmt6 mutants were constructed by the integration of two homologous regions using uracil primers into the GU2 template vector using the USER friendly cloning technique. The vectors were introduced to spores from the p∆kmt6 strain by ATMT.

### 4.5. NMR

All of the NMR experiments were recorded on a BRUKER AVIII-600 MHz NMR spectrometer that was equipped with a CPP-TCI cryogenically cooled probe. Spectra were recorded and processed with TopSpin 3.5pl6. The compound was dissolved in DMSO-d6, and the following NMR spectra were recorded at 308.1 K: ^1^H-NMR, [^1^H,^13^C]-HSQC, [^1^H,^13^C]-HMBC optimized for *J* = 8 Hz, 2D-TOCSY with 100 ms MLEV17 mixing at γB_1_/2π = 10 kHz, 2D-ROESY with a 250 ms spin-lock at γB_1_/2π = 5916 Hz, DQF-COSY, and a [^1^H,^15^N]-HSQC. All of the spectra were referenced to the resonance of DMSO (^1^H: 2.5 ppm, ^13^C: 39.51 ppm). ^15^N chemical shifts were indirectly referenced using Ξ = 0.10132912. The assignment and chemical shift of ^13^C were reviewed by CSEARCH Robot [35].

### 4.6. Whole-Genome Sequencing Validation of Mutants/Southern by Sequencing

Illumina Sequencing of OE::*NRPS5, OE::NRPS9, ΔNRPS9,* and OE::*NRPS5::NRPS9*. Genomic DNA from 5 mg freeze-dried mycelium was extracted with the FastDNA SPIN Kit for Soil (MP Biomedicals, Fisher Scientific, Roskilde, Denmark) and subsequently cleaned with Agencourt AMPure XP beads (Beckman Coulter, Ramcon, Birkerød, Denamrk) using a 1.7 bead:sample ratio. A Nextera paired-end DNA library (Illumina) was prepared according to the Nextera DNA Library Prep Reference Guide (Document # 15027987 v01), with the modification of using Agencourt AMPure XP beads (0.7 bead:sample ratio) for the post-tagmentation clean-up. DNA quality (A260/A280 and A260/A230), size-distributions, and concentration were assessed on the NanoDrop ND-1000 (Thermo Scientific), D1000 ScreenTape system (Agilent, Glostrup, Denmark), and using the Qubit dsDNA HS assay kit (Thermo Scientific), respectively. Each paired-end library was sequenced to approximately 200× coverage on the Illumina HiSeq 2500 system while using the Rapid SBS Kit v2 (2 × 250 cycles). Low quality reads, ambiguous nucleotides, and adaptors were trimmed (custom trim settings) in CLC Genomics Workbench v. 9.4.2 (Qiagen, Vedbæk, Denmark). Reads were subsequently mapped to the corresponding mutant reference genome in CLC Genomics Workbench while using the default settings. High-molecular weight (HMW) genomic DNA were extracted from 250 mg freeze-dried mycelia by incubation in 10 mL Qiagen lysis buffer C2 (Qiagen) for 2 h at 50 °C and in the presence of 10 mg RNase A (Qiagen) and 6 mg Proteinase K (Qiagen). Following two rounds of 1:1:1 (*v*/*v*) chloroform:phenol:isoamyl extraction (Sigma-Aldrich, Søborg, Denmark), HMW DNA was precipitated with 1:1 (*v*/*v*) room-temperature isopropanol. DNA was spooled into new tubes and washed twice with 70% ethanol. Following the removal of residual ethanol, DNA was allowed to re-dissolve in 10 mM Tris pH 8.5 for a minimum of 48 h. DNA concentration, quality, and size-distribution were assessed, as previously described. Approximately 400 ng of HMW DNA was used as the input for the Rapid Barcoding kit protocol (SQK-RBK004) (Oxford Nanopore Technologies, Oxford, UK). Sequencing was carried out on a R9.4 FLO-MIN106 flow-cell in the MinION sequencer (Oxford Nanopore Technologies), with approximately 10–30× coverage. Base-calling and barcode sorting was performed in Albacore v. 2.2.7. The reads were subsequently mapped to the corresponding mutant reference genome in CLC Genomics Workbench.

### 4.7. Pathogenicity Assays

For *F. graminearum* infections, the highly susceptible (FHB) USU-Apogee full-dwarf hard red spring wheat (Triticum aestivum cv. USU-Apogee; Reg.no CV-840, PI592742) was used. Type II infections were carried out by inoculating two spikelets per wheat ear with 10 μL of a 10^5^ spores/mL solution. As a control, the MOCK ears were inoculated with water instead of the spore suspension. Five ears per *F. graminearum* strain were inoculated, as well as five MOCK ears. After each treatment, the ears were covered in moistened plastic bags for the first 24 h to provide high humidity. The incubation conditions were set to 60% humidity, 20 °C for 16 h (day) and 18 °C for 6 h (night). The *F. graminearum* infection progress was monitored by counting the infected spikelets over a time period of 10 days.

### 4.8. Metabolite Extraction and Analytical LCMS/bbCID of Fusaoctaxin A’s

The secondary metabolites were extracted from Wt *F. graminearum*, OE::*NRPS5*, OE::*NRPS9,* and OE::*NRPS5OE::NRPS9* that were grown in triplicates for two weeks at 25 °C in the dark on solid yeast extract sucrose (YES) media in 90 mm petri dishes [36]. Fifteen plugs (3 mm) from each sample was covered with 5 mL extraction solvent A (*v*:*v*:*v* 3:2:1 ethyl acetate:dichloromethane:methanol (HiPerSolv CHROMANORM, VWR, Søborg, Denmark) with 1% formic acid (95%, Sigma-Aldrich) and sonicated for 45 min. The extracts were decanted into new tubes and dried under a flow of N_2_ gas. The dried samples were re-dissolved in 400 µL methanol and centrifuged for 5 min. at 14.1k rcf. The supernatants were transferred to 2 mL HPLC vials. The samples were analyzed for secondary metabolites by HPLC-DAD-HRMS while using a Hitachi Elite LaChrom HPLC system, which was equipped with a C6-phenyl (150 × 4.6 mm Ascentis Xpress 2.7 μm, Sigma-Aldrich) column kept at 40 °C and coupled by a 5:95 flow splitter to a high-resolution mass spectrometer (compact qTOF, Bruker) with an electrospray source (Capilary: 4500 V; end plate offset 500 V; Dry gas 4.0 L/min, 200 °C) that was operated in positive mode. The injection volume was set to 20 µL and separated using a 1 mL/min gradient initiating at 10% solvent A (acetonitrile (HiPerSolv CHROMANORM, VWR) with 0.1% *v*/*v* formic acid (MS-grade 98%, Sigma-Aldrich)) in 90% solvent B (water (HiPerSolv CHROMANORM, VWR) with 0.1% *v*/*v* formic acid (MS-grade 98%, Sigma-Aldrich)), increasing linearly to 100% solvent A over 20 min. and then held for 10 min. After 0.2 min. from sample injection, 2 µL calibrant (10 mM NaOH and 26 mM formic acid (MS-grade 98%, Sigma-Aldrich) in 1:1 water (HiPerSolv CHROMANORM, VWR):isopropanol) was directly injected to the mass analyzer and was used for internal spectra calibration. Mass spectrometric instrument control, data recording, and data processing were performed using Compass DataAnalysis 4.2 SR2 (Bruker). The metabolite extract from OE::NRPS5::NRPS9 was additionally analysed by bbCID fragmentation that was acquired at 20 eV.

### 4.9. Metabolite Extraction and Analytical HPLC-HRMS of ABC Transporter and Peptidase KO Mutants

Wild type *F. graminearum*, OE::*NRPS5*::*NRPS9*, Δ*kmt6*, Δ*fgm5*+Δkmt6, and ΔFGSG_10982+Δ*kmt6* were grown in triplicates on solid YES agar medium for 12 days at 25 °C in the dark. The metabolites were extracted from six plugs that were covered with 3 mL extraction solvent A and sonicated for 40 min., otherwise following same procedure, as previously described. HPLC-HRMS analysis was acquired, as previously described, except the gradient was held at 100% solvent A for 5 min.

### 4.10. Metabolite Extraction and Analytical HPLC-HRMS of Fusarium Infected Barley

Barley that was infected with wild type *F. graminearum*, OE::*NRPS5*, OE::*NRPS9*, OE::*NRPS5*:*NRPS9*, Δ*NRPS9,* and a MOCK were analyzed for secondary metabolites. Extraction was performed on approx. 0.2 g grinded barley powder using 1 mL of acetonitrile:water (82:18 *v*/*v*). The samples were gently mixed and sonicated for 25 min. The solvent was centrifuged at 14.1k rfc and transferred to a 2 mL HPLC vial. HPLC-HRMS analysis was performed, as described above.

### 4.11. Purification of Fusaoctaxin A and Fusatrixin A

Fusaoctaxin A was purified from OE::*NRPS5::NRPS9* that was grown on 25 YES agar plates at 25 °C in the dark for 14 days. The fungi were diced to roughly 5X5 mm and covered with 400 mL of extraction solvent A and sonicated for 45 min. The solvent was filtered through Miracloth (Merck) and subsequently evaporated using a rotary evaporator at 40 °C. The dried metabolites were re-dissolved in 1 mL methanol (HiPerSolv CHROMANORM, VWR), centrifuged at 14.1k rcf, and then transferred to a 2 mL HPLC vial. Fusatrixin A was purified from 120 YES agar plates following the same procedure as for fusaoctaxin A, except 1.2 L of ethyl acetate (HiPerSolv CHROMANORM, VWR) with 1% formic acid (95%, Sigma-Aldrich) was used as the extraction solvent and sonication was performed for 1 h. The dried metabolites were re-dissolved in 8 mL methanol (HiPerSolv CHROMANORM, VWR) and subsequently reduced to 2.5 mL by a flow of nitrogen gas. The semi-preparative HPLC was conducted on an Agilent 1260 infinity LC system (Agilent Technologies) with a diode array detector (190–900 nm). 100 µL sample was injected and separated on a 150 × 10.0 mm Gemini 5 µm C6-Phenyl 110 Å column (Phenomenex, Værløse, Denmark)using a flow of 5 mL/min and with a linear gradient of water (MilliQ)-acetonitrile (HiPerSolv, VWR), with both being supplemented with 50 ppm trifluoroacetic acid (Sigma-Aldrich). The gradient initiated at 10% acetonitrile increasing to 100% over 12 min. and held for 2 min. before returning to initial conditions over the final 2 min. The peaks of interest were detected by UV at 195 nm and then collected.

### 4.12. Marfey’s Reaction and HPLC-HRMS

Fusaoctaxin A was quantified by a PULCON NMR experiment in DMSO-d_6_ to 250 µg. Full hydrolysis was performed in 2.4 mL of 1:3 DMSO-d_6_:8M HCl at 110 °C for 24 h under a nitrogen atmosphere. The hydrolyzed sample was diluted 1:10 with Milli-Q water, flash-frozen in liquid nitrogen, and lyophilised. The dried sample was re-dissolved in 200 µL Milli-Q water and derivatized with N-α-(2,4-dinitro-5-fluorophenyl)-l-alaninamid (Sigma-Aldrich), together with commercially bought amino acid standards (d-Ala, l-Ala, d-allo-Ile, l-Ile, d-ser, l-ser, d-Val, l-Val, d-Leu, l-Leu, d-Leucinol, l-Leucinol) [37]. The derivatized amino acids were separated on a C18 (150 × 4.6 mm Ascentis Xpress 2.7 μm, Sigma-Aldrich) column by HPLC-HRMS while using a gradient starting at 10% solvent A linearly increasing to 40% over 45 min., then increased to 99% solvent A, and held for 5 min.

### 4.13. Bioactivity

We used the OcelloScope optical detection system (BioSense Solutions, Farum, Denmark) [38,39] to monitor the growth of *Fusarium graminearum* and *Fusarium Solani* exposed to chemical synthesized fusaoctaxin A, fusapentaxin A, and fusatrixin A in the concentration range 0–100 µM in real-time. Briefly, growth was monitored at 25 °C in Nunc Edge 2.0 96-Well plates (Thermo Fischer Scientific) containing 200 µL sterile-filtered YPG media (10 g·L^−1^ yeast extract, 20 g·L^−1^ peptone, and 50 mL·L^−1^ sterile-filtered 50% (*w*/*v*) d-(+)-glucose), 5000 spores and 0 µM (0.5% ethanol control), 12.5 µM, 25 µM, 50 µM, or 100 µM fusaoctaxin A’s. Sterile medium (8 mL) was added to the edge-reservoirs to minimize the sample evaporation. Focus and illumination level was manually adjusted following a 0.5 h spore settling-time. Two image-strips (11.43 µm image distance) per. well were automatically acquired in UniExplorer version 7.3.0.1057 (20 min. intervals and 180 repetitions) (Biosense Solutions, Farum, Denmark).

## Supplementary Materials

The following are available online at https://www.mdpi.com/2072-6651/11/5/277/s1, Figure S1: Synteny map of the fusaoctaxin A gene cluster, Figure S2: Southern by sequencing, Figure S3: HPLC-HRMS analysis of secondary metabolite extracts, Figure S4: NMR Spectroscopic Data, Figure S5: Region of TOCSY NMR spectrum of fusaoctaxin A, Figure S6: Marfey’s assay for determination of absolute configuration of amino acids from fusaoctaxin A, Figure S7: Amino acid sequence validation for compounds using 20 eV broadband Collision Induced Dissociation (bbCID) spectra, Figure S8: Hierarchical clustering, Figure S9: No phenotypic effects of fusaoctaxin A, fusapentaxin A and fusatrixin A in Fusarium, Table S1: Primer designs for gene knockout and over-expression mutants.
