# Supplementary material for: Fusaoctaxin A, an Example of a Two-Step Mechanism for Non-Ribosomal Peptide Assembly and Maturation in Fungi
Source: Toxins (Basel). 2019 May 16;11(5):277. doi: 10.3390/toxins11050277 (PMC6563249; doi:10.3390/toxins11050277)
Supplement: Supplementary file 1 [file toxins-11-00277-s001.pdf]

Supplementary Materials: Fusaoctaxin A, An Example of a Two-Step Mechanism for Non-Ribosomal Peptide Assembly and Maturation in Fungi

Klaus Ringsborg Westphal, Katrine Amalie Hamborg Nielsen, Rasmus Dam Wollenberg, Mathias Bonde Møllehøj, Simone Bachleitner, Lena Studt, Erik Lysøe, Henriette Giese, Reinhard Wimmer, Jens Laurids Sørensen and Teis Esben Sondergaard *

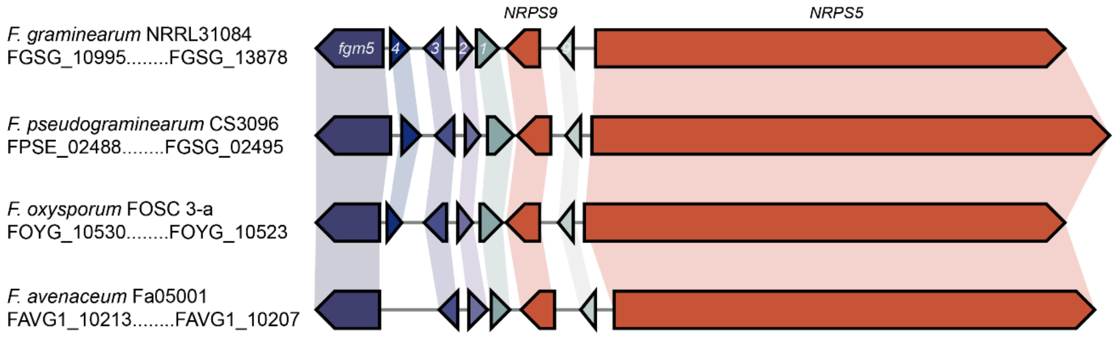

Figure 1. Synteny map of the fusaoctaxin A gene cluster. Clusters from *F. graminearum*, *F. pseudograminearum*, *F. oxysporum* and *F. avenaceum* were predicted from Antismash 3.0. An orthologue of the ankyrin-rich protein *fgm4* is present in *F. avenaceum*, but was not annotated in the original published genome sequence.

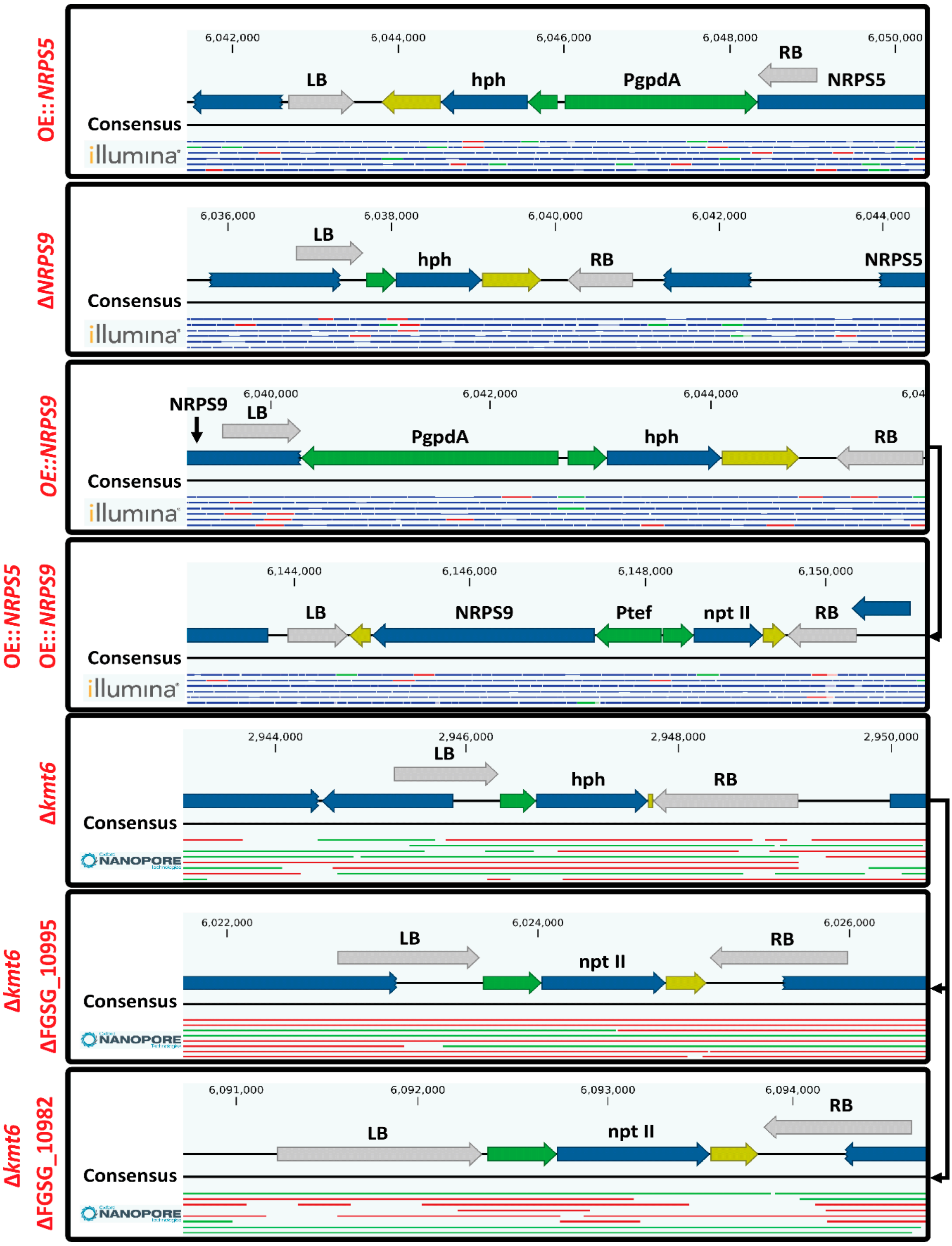

Figure 2. Southern by sequencing. Validation of mutant strains by either short-read Illumina sequencing or Oxford Nanopore long-read sequencing. The representative read-mappings are depicted as either paired-end (blue), sense strand (green) or anti-sense strand (red) reads. Only relevant annotations are highlighted. Green, yellow and blue annotations represent promotors, terminators and genes. Left (LB) and right (RB) borders indicate regions used to facilitate the targeted double homologous recombination event. A blastN analysis of contigs from a *de novo* assembly of Illumina reads and long-reads resulting from Oxford

Nanopore sequencing were conducted to confirm single integration events of the *hph* (hygromycin B phosphotransferase) or *nptII* (Neomycin phosphotransferase) genes. PgpdA: glyceraldehyde-3-phosphate dehydrogenase promoter, Ptef: Translation elongation factor 1-alpha promotor.

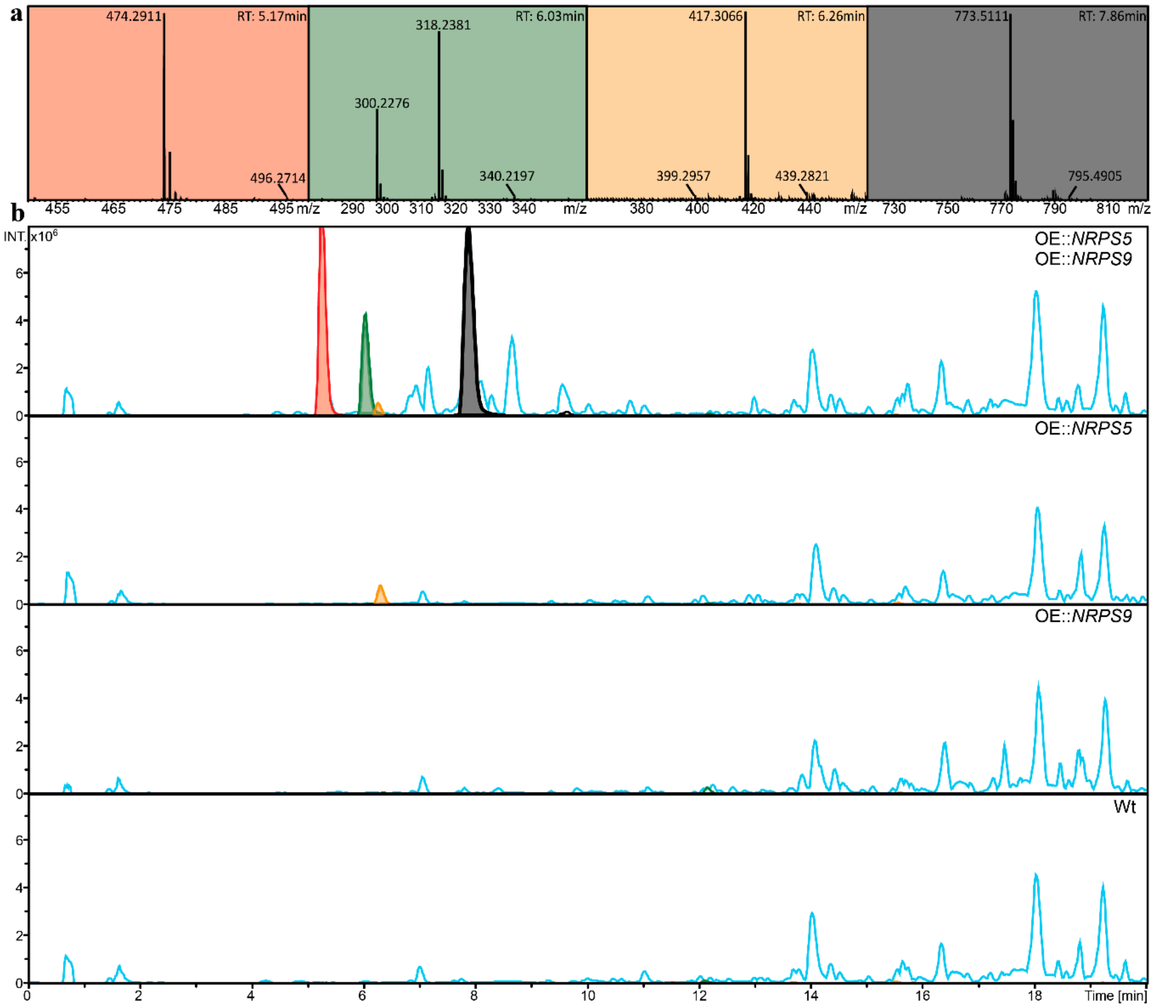

Figure S3. HPLC-HRMS analysis of secondary metabolite extracts from OE::*NRPS5*OE:: *NRPS9*, OE:: *NRPS5*, OE:: *NRPS9*, and wild type grown on rice agar medium. **a**, Zooms of four mass spectra from OE::*fgrA*OE::*fgrB*. Red: RT 5.17 min. showing fusapentaxin A $[M+H]^+$ (*m*/*z* 474.2911) and $[M+Na]^+$ (*m*/*z* 496.2714). Green: RT 6.03 min. showing fusatrixin A $[M+H]^+$ (*m*/*z* 318.2381), $[M+Na]^+$ (*m*/*z* 340.2197) and $[M-H_2O+H]^+$ (*m*/*z* 300.2276). Orange: RT 6.26 min. showing fusatetraxin A $[M+H]^+$ (*m*/*z* 417.3066), $[M+Na]^+$ (*m*/*z* 439.2821) and $[M-H_2O+H]^+$ (*m*/*z* 399.2957). Black: RT 7.86 min. showing fusaoctaxin A $[M+H]^+$ (*m*/*z* 773.5111) and $[M+Na]^+$ (*m*/*z* 795.4905). **b,** Four BPCs (blue) with EICs at *m*/*z* 474.2911 ± 0.5 Da (red), 318.2381 ± 0.5 Da (green), 417.3066 ± 0.5 Da (orange) and 773.5111 ± 0.5 Da (black).

(a)

**A**				Fusaoctaxin A			
Residue	Atom	δ_C, type	Atom	δ_H (J in Hz)	δ_N	HMBC[a]	ROESY
1 GABA	C	171.06, C	NH_2	7.62 (Brd)	117.1		
	C^{α}	31.7, CH_2	H^{α}	2.22 (ddd, 2.2, 8,3)		$1C^{\beta}$, $1C^{\gamma}$, 1C	2NH
	C^{β}	23.0, CH_2	H^{β}	1.75 (m, 2.2, 7,2)		$1C^{\alpha}$, $1C^{\gamma}$, 1C	
	C^{γ}	38.5, CH_2	H^{γ}	2.80 (Brd dd, 7.2)		$1C^{\beta}$	
2 L-ALA	C	172.4, C	NH	8.08 (d, 7.3)	125.8	$2C^{\beta}$, $2C^{\alpha}$, 1C	$1H^{\alpha}$, 3NH
	C^{α}	48.2, CH	H^{α}	4.41[b]		$2C^{\beta}$, 2C	3NH
	C^{β}	18.5, CH_3	H^{β}	1.20 (d, 7,0)		$2C^{\alpha}$, 2C	3NH
3 D-Ile	C	171.1, C	NH	7.845[b]	111.2	$3C^{\alpha}$, 2C	$2H^{\alpha}$, $2H^{\beta}$, 2NH, 4NH
	C^{α}	55.02, CH	H^{α}	4.42[b]		$3C^{\gamma 2}$,$3C^{\beta}$, $3C^{\gamma 1}$, 3C	4NH
	C^{β}	37.1, CH	H^{β}	1.84 (m, 6.6)		$3C^{\gamma 1}$, $3C^{\gamma 2}$, $3C^{\delta}$	4NH
	$C^{\gamma 1}$	25.8, CH_2	$H^{\gamma 1a}$	1.08 (m)		$3C^{\delta}$, $3C^{\gamma 2}$, $3C^{\beta}$, $3C^{\alpha}$	
			$H^{\gamma 1b}$	1.30[b]		$3C^{\delta}$, $3C^{\gamma 2}$, $3C^{\beta}$, $3C^{\alpha}$	
	$C^{\gamma 2}$	14.2, CH_3	$H^{\gamma 2}$	0.77 (d, 6.8)		$3C^{\gamma 1}$, $3C^{\beta}$, $3C^{\alpha}$	
	C^{δ}	11.5, CH_3	H^{δ}	0.84b		$3C^{\gamma 1}$, $3C^{\beta}$	
4 D-Ser	C	169.9, C	NH	8.01 (d, 7.5	115.4	$4C^{\alpha}$, $4C^{\beta}$, 3C	$3H^{\alpha}$, $3H^{\beta}$, 3NH, 5NH
	C^{α}	55.03, CH	H^{α}	4.38 (dd, 5.8, 7.5)		$4C^{\beta}$, 4C, 3C	5NH
	C^{β}	61.4, CH_2	$H^{\beta 2,3}$	3.60 (ddd, 5.6)		$4C^{\alpha}$, 4C	5NH
			OH	4.89 (dd, 5.3)			
5 D-Val	C	170.6, C	NH	7.66 (d, 8.6)	115.6	$5H^{\alpha}$, 4NH, 6NH	$4H^{\alpha}$, $4H^{\beta}$, 4NH, 6NH
	C^{α}	57.4, CH	H^{α}	4.22[b]		$5C^{\gamma 1}$,$5C^{\gamma 2}$,C^{β}, 5C, 4C	6NH
	C^{β}	30.5, CH	H^{β}	2.00 (m, 6.6)		$5C^{\gamma 1}$, $5C^{\gamma 2}$, $5C^{\alpha}$, 5C	6NH
	$C^{\gamma 1}$	17.6, CH_3	$H^{\gamma 1}$	0.82[b]		$5C^{\gamma 2}$, $5C^{\beta}$, $5C^{\alpha}$	6NH
	$C^{\gamma 2}$	19.0, CH_3	$H^{\gamma 2}$	0.85[b]		$5C^{\gamma 1}$, $5C^{\beta}$, $5C^{\alpha}$	6NH
6 D-Ser	C	169.7, C	NH	7.92 (d, 7.7)	115.2	$6C^{\alpha}$, 5C	$5H^{\alpha}$, $5H^{\beta}$, $5H^{\gamma 1}$, $5H^{\gamma 2}$, 5NH, 7NH
	C^{α}	54.6, CH	H^{α}	4.32 (ddd, 6.6, 7.3)		$6C^{\beta}$, 6C, 5C	7NH
	C^{β}	61.6, CH_2	$H^{\beta 2}$	3.50 (ddd,		6C	
			$H^{\beta 3}$	3.57 (ddd, 4.6, 4.8, 5.5)		6C	
			OH	5.06 (5.5, 4.8)			
7 D-Leu	C	171.2, C	NH	7.842[b]	120.9	$7C^{\alpha}$, 6C	$6H^{\alpha}$, 6NH, 8NH
	C^{α}	51.3, CH	H^{α}	4.23[b]		$7C^{\beta}$, 7C, 6C	8NH
	C^{β}	40.6, CH_2	$H^{\beta 2,3}$	1.42-1.50[b]		$7C^{\gamma}$, $7C^{\delta 1}$,$7C^{\delta 2}$, $7C^{\alpha}$, 7C	
	C^{γ}	24.1, CH	H^{γ}	1.60 (m, 6.6, 7.0)		$7C^{\delta 2}$, $7C^{\delta 1}$, $7C^{\beta}$	
	$C^{\delta 1}$	21.3, CH_3	$H^{\delta 1}$	0.82[b]		$7C^{\delta 2}$, $7C^{\gamma}$, $7C^{\beta}$	
	$C^{\delta 2}$	23.0, CH_3	$H^{\delta 2}$	0.86[b]		$7C^{\delta 1}$, $7C^{\gamma}$, $7C^{\beta}$	
8 L-Leucinol	C	63.8, CH_2	H^{1}	3.18 (ddd, 6.0)	120.5	$8C^{\alpha}$, 7C	$7H^{\alpha}$, 7NH
			H^{2}	3.28[b]		$8C^{\beta}$, $8C^{\alpha}$,	
	C^{α}	48.7, CH	H^{α}	3.75 (m, 4.7, 5.4)		$8C^{\beta}$, $8C^{\alpha}$,	
	C^{β}	39.8, CH_2	$H^{\beta 2,3}$	1.27[b]		8C	
	C^{γ}	24.0, CH	H^{γ}	1.53[b]		$8C^{\delta 1}$, $8C^{\delta 2}$, $8C^{\gamma}$, $8C^{\alpha}$, 8C	
	$C^{\delta 1}$	21.6, CH_3	$H^{\delta 1}$	0.80[b]		$8C^{\beta}$	
	$C^{\delta 2}$	23.3, CH_3	$H^{\delta 2}$	0.85[b]		$8C^{\delta 2}$, $8C^{\gamma}$, $8C^{\beta}$	
			NH	7.42 (d, 8.7)		$8C^{\delta 1}$, $8C^{\gamma}$, $8C^{\beta}$	

[a]HMBC correlations, optimized for long range J_{CH} of 8 Hz, are from proton(s) stated to the indicated carbon.
[b]Signal overlapping

b

(b)

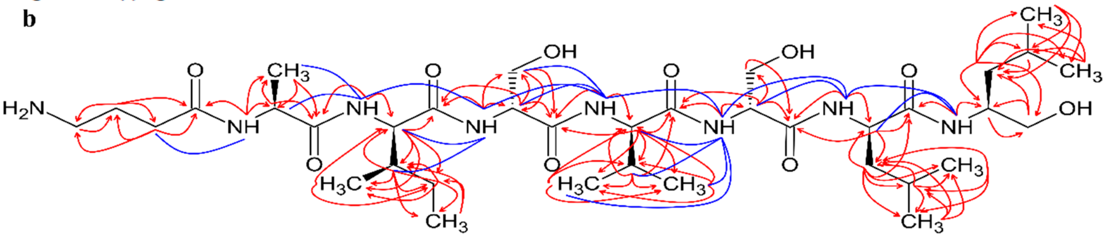

Figure S4. NMR Spectroscopic Data (600 MHz, $(CD_3)_2SO$) for fusaoctaxin A. **a,** Chemical shifts from the eight residues (1-8) for carbon (δ_C) including multiplicity (type), for hydrogen (δ_H) including coupling constants (J) in Hz where possible and for nitrogen (δ_N). HMBC correlations, optimised for long range J_{CH} of 8 Hz, are from proton(s) stated to the indicated carbon. ^1H-^1H ROESY correlations only for inter-residue connectivities. [1]Partial or full overlapped signal. **b,** Structure of fusaoctaxin A elucidated by NMR with ^1H-^{13}C HMBC (red arrows; directionality H→C) and inter-residue ^1H-^1H ROESY (blue lines) correlations.

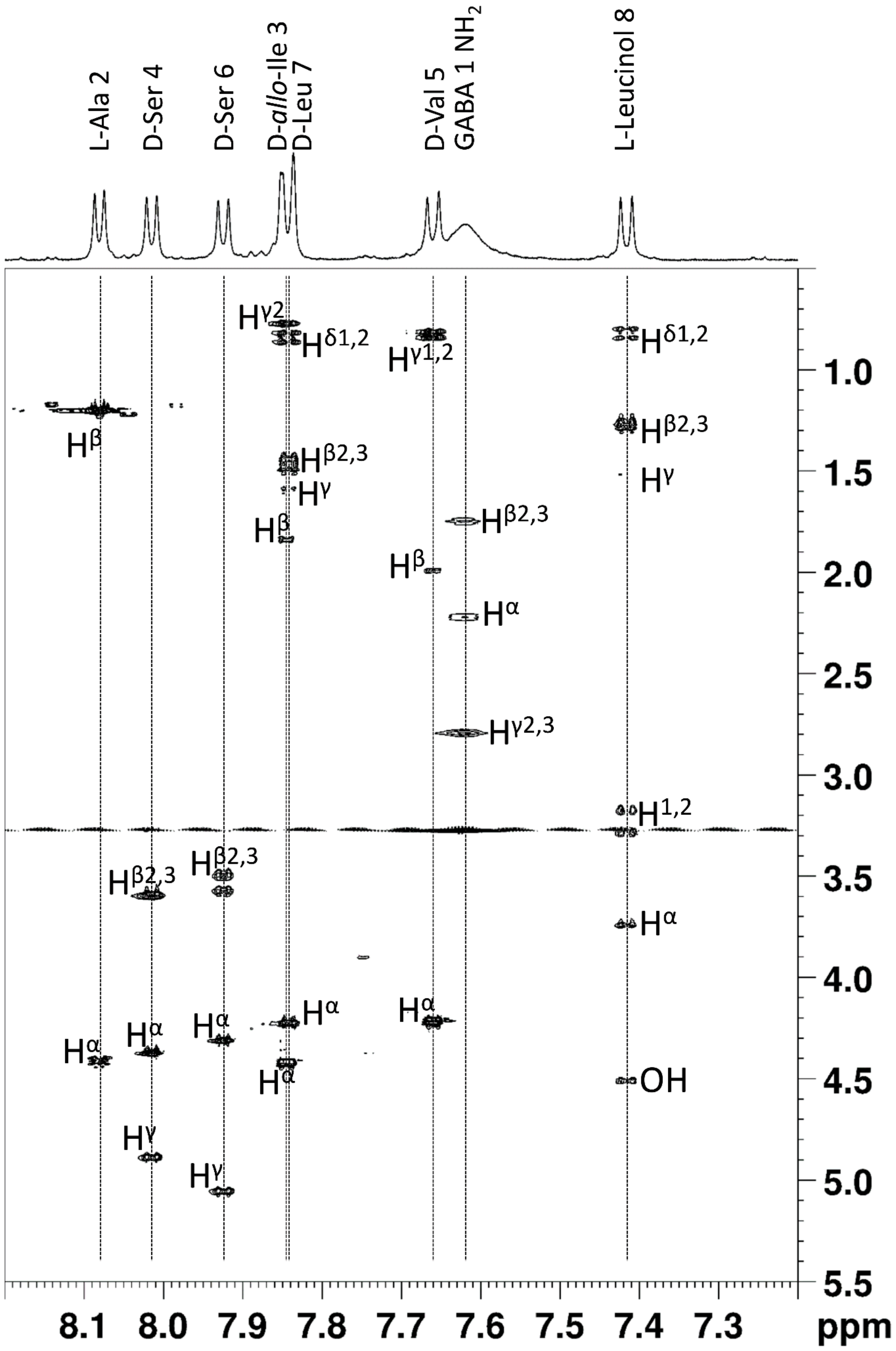

Figure S5. Region of TOCSY NMR spectrum of fusaoctaxin A in DMSO-d6. Eight spin systems (1–8) of fusaoctaxin A showing correlations (dotted lines) from H^N to side chain atoms.

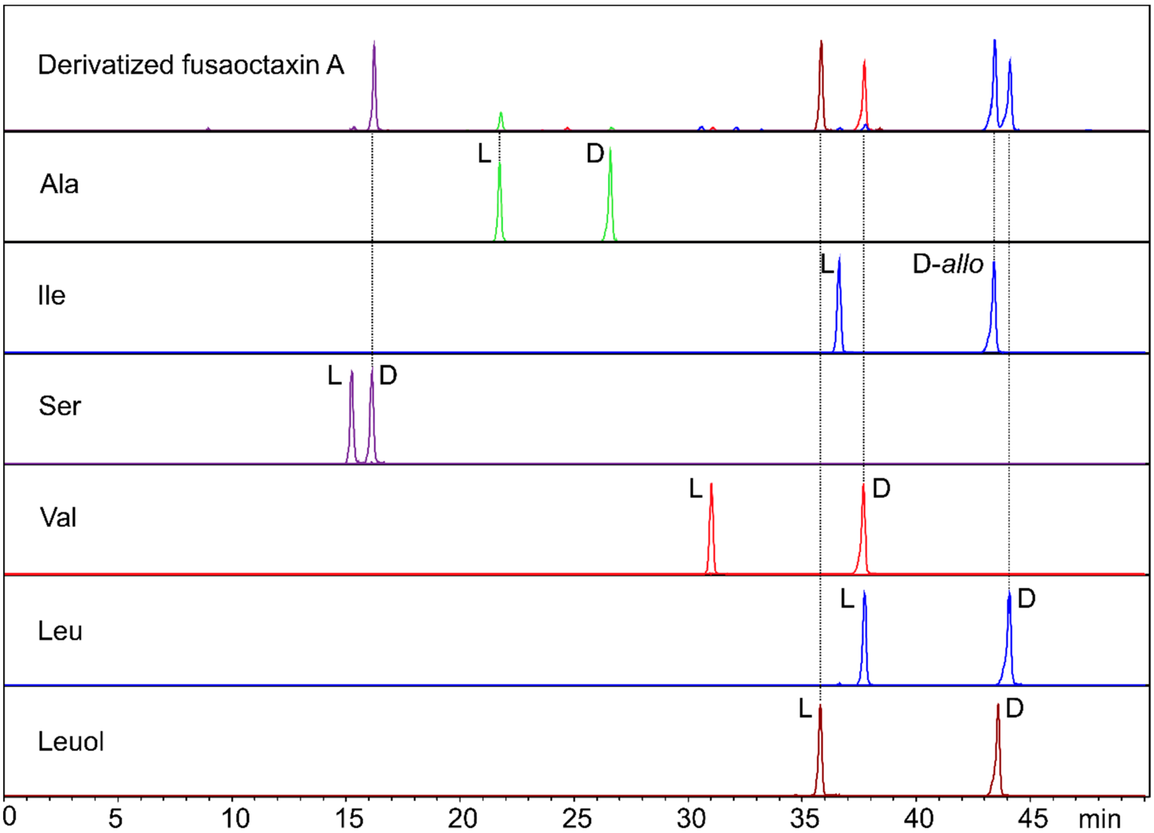

Figure S6. Marfey's assay for determination of absolute configuration of amino acids from fusaoctaxin A. Extracted ion chromatograms (EICs) from derivatised fusaoctaxin A aligned with EICs of derivatised L- and D- amino acid standards. L- and D- standards were analysed separately and the EICs have subsequently been overlaid. Dotted vertical lines indicate alignment from fusaoctaxin A to the respective standard. Green: EIC *m*/*z* 342.1042 ± 0.005 Da (derivatised alanine), Blue: EIC *m*/*z* 384.1514 ± 0.005 Da (derivatised isoleucine/leucine), Purple: EIC *m*/*z* 358.0993 ± 0.005 Da (derivatised serine), Red: EIC *m*/*z* 370.1357 ± 0.005 Da (derivatised valine), Brown: EIC *m*/*z* 370.1721 ± 0.005 Da (derivatised leucinol).

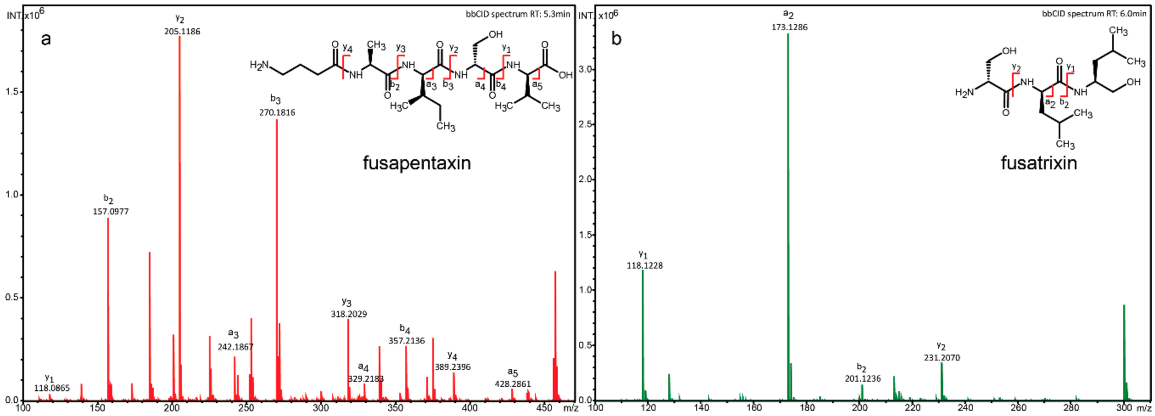

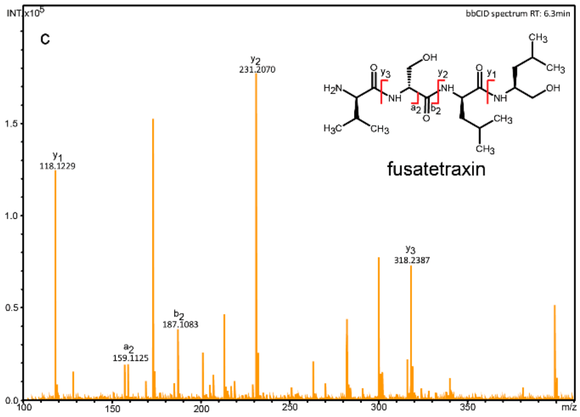

d

	Ion	INT. %	Measured M	Theoretical M	Δ Mass PPM
fusapentaxin	Y1	0.4	118.0865	118.0863	1.69
	b2	11.1	157.0977	157.0972	3.18
	Y2	22.2	205.1186	205.1183	1.46
	a3	2.7	242.1867	242.1863	1.65
	b3	17.1	270.1816	270.1812	1.48
	Y3	5.0	318.2029	318.2023	1.89
	a4	1.1	329.2183	329.2183	0.00
	b4	3.4	357.2136	357.2132	1.12
	Y4	1.7	389.2396	389.2395	0.26
	a5	0.7	428.2861	428.2867	-1.40
fusatrixin	y1	35.6	118.1228	118.1226	1.69
	a2	100.0	173.1286	173.1285	0.58
	b2	4.4	201.1236	201.1234	0.99
	y2	10.4	231.207	231.2067	1.30
fusatetraxin	y1	70.3	118.1229	118.1226	2.54
	a2	11.0	159.1125	159.1128	-1.89
	b2	21.7	187.1083	187.1077	3.21
	y2	100	231.207	231.2067	1.30
	y3	41.2	318.2387	318.2387	0.00

Figure S7. Amino acid sequence validation for compounds using 20 eV broadband Collision Induced Dissociation (bbCID) spectra from a HPLC-HRMS analysis of an OE::*NRPS5*OE::*NRPS9* metabolite extract. **a,** bbCID spectrum of fusapentaxin (RT: 5.3 min, $[M+H]^+$ 474.2911 Da). The chemical structure of fusapentaxin with indicated a-, b-, y- fragmentations. **b,** bbCID spectrum of fusatrixin (RT: 6.0 min, $[M+H]^+$ 318.2381 Da). The chemical structure of fusatrixin with indicated a-, b-, y- fragmentations. **c,** bbCID spectrum of fusatetraxin (RT: 6.3 min, $[M+H]^+$ 417.3066 Da). The chemical structure of fusatetraxin with indicated a-, b-, y- fragmentations. **d,** Table of detected a-, b-, y- fragment ions for fusapentaxin (red), fusatrixin (green), and fusatetraxin (orange), including relative intensity (INT. %), the theoretical charged monoisotopic mass (theoretical M), and the calculated mass deviation (PPM).

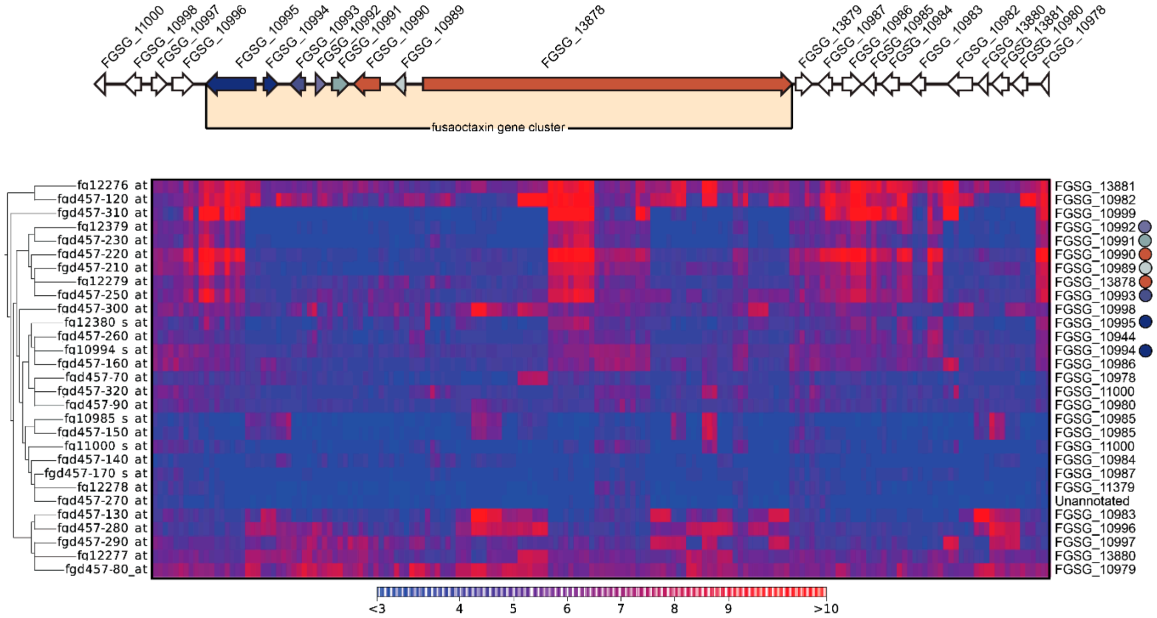

Figure S8. Hierarchical clustering. **a,** Fusaoctaxin A gene cluster in *F. graminearum*. **b,** 176 *F. graminearum* microarray chips from different experiments, using 29 probes representing fusaoctaxin A gene cluster and flanking genes.

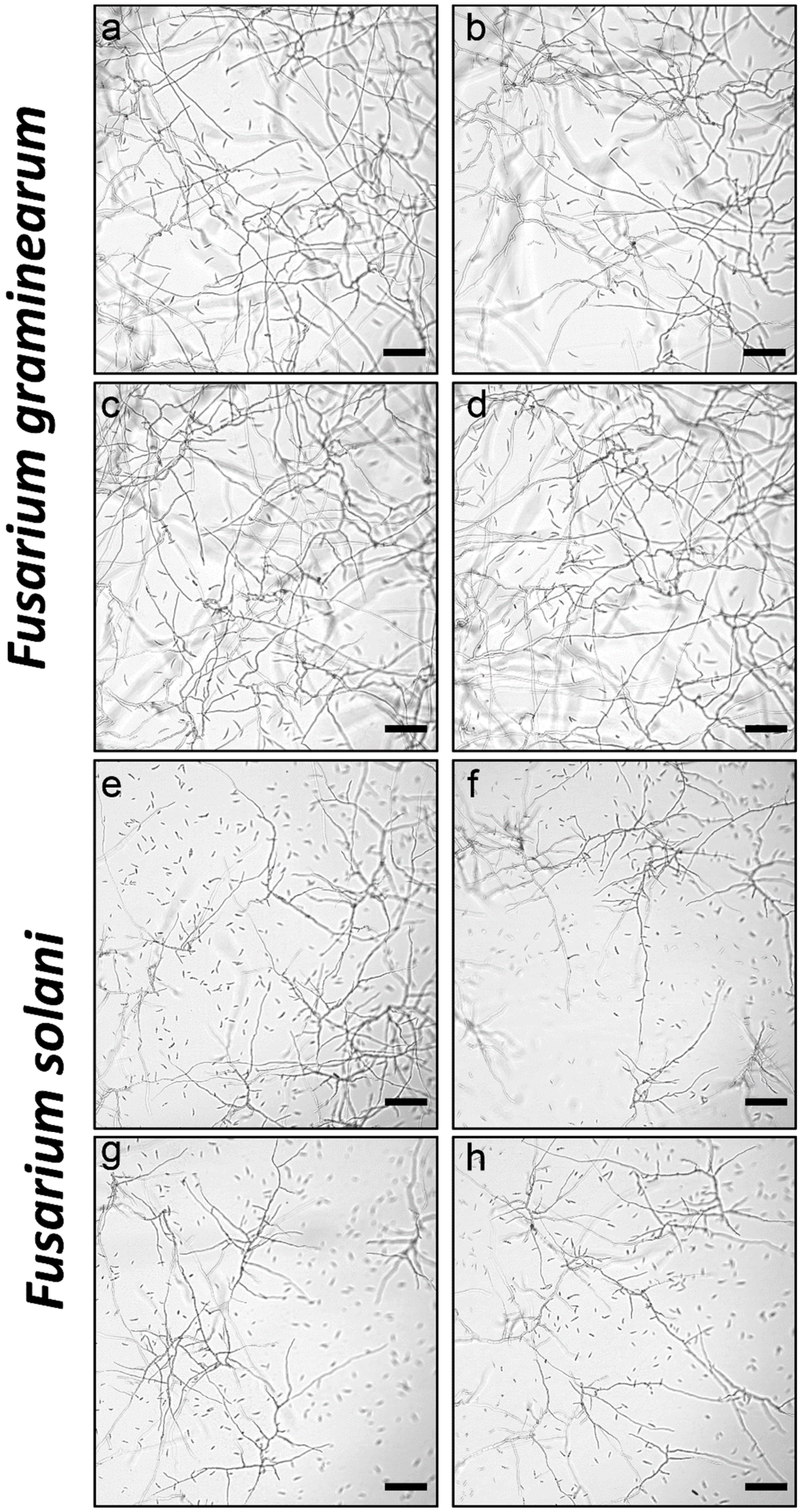

Figure S9. No phenotypic effects of fusaoctaxin A, fusapentaxin A *and* fusatrixin A in *Fusarium*. **a, e,** Control with 0.5% ethanol. **b, f,** 100 μM fusaoctaxin A. **c, g,** 100 μM fusapentaxin A. ***d, h,*** 100 μM fusatrixin A. All experiments were performed in triplicates, scale bar is 200 μm.

Table S1. Primer designs for gene knockout and over-expression mutants. [U] Primers designed with 9 bp overhangs for USER friendly cloning.

Locus Tag	Oligo Name	Description	Sequence (5′ → 3′)
**FGSG_10982**	O1	Peptidase KO	GGTCTTAA[U]CGACGTACAGATTACTTGCCAC
	O2		GGCATTAA[U]GAGACTGTTGGGGAGAGTGATA
	A3		GGACTTAA[U]TAGATCATGCAGGTGTGCTATC
	A4		GGGTTTAA[U]CGTAGTTATTGGTCAAGACAGG
**FGSG_10995**	O1	ABC transporter KO	GGTCTTAA[U]CAGTGGCGAAGCAGTCTGAA
	O2		GGCATTAA[U]ACAGTAGCCAGTCATCGGTCAA
	A3		GGACTTAA[U]TATCCAACGGCAGGCAGCTAT
	A4		GGGTTTAA[U]AGTTCGTCCTTGCCATTGACG
**FGSG_10990**	O1	NRPS9 KO	GGTCTTAA[U]GGAGGCCGTTTTGTATGGTACCAG
	O2		GGCATTAA[U]CAGATAGAAGAGACACAATCCAGTTGCG
	A3		GGACTTAA[U]TCTTGTCTTCAATATCGGTGCTGTCT
	A4		GGGTTTAA[U]TTTGAACAAGGCCCCTATTCTCGAA
**FGSG_10990**	O1	NRPS9 OE	GGTCTTAA[U]GGAGGCCGTTTTGTATGGTACCAG
	O2		GGCATTAA[U]CAGATAGAAGAGACACAATCCAGTTGCG
	O3		GGACTTAA[U]GGCTCCTCTTAACACTTATACGTCTACT
	O4		GGGTTTAA[U]TCGTTGAGCTGATGGTACGTCAAATT
**FGSG_17487**	O1	NRPS5 OE	GGTCTTAA[U]GGTGTGATGTGTATAAATGAATGTGAATGTG
	O2		GGCATTAA[U]TAGATGATAACAATAGTATGGTAATGATGTGTGTG
	O3		GGACTTAA[U]GCCGCCACAAGATCATCCTA
	O4		GGGTTTAA[U]CGAGCACGCTGGGCAATCAT
**FGSG_10990**	Fw	NRPS9 OE (tubulin)	ATTTCCC|GGGATGGCTCCTCTTAACACT
	Rv		ATTTGG|CGCGCCCTATAAAACAGTAACTGTAACCTG
**FGSG_15795**	5F	Kmt6 KO	AACGCCAGGGTTTTCCCAGTCACGACGCACCCTTCTTGGATGACTCGC
	5R		ACTTAACGTTACTGAAATCTCCAACGTGCAGGACTGCAGTTGTCAAGG
	3F		TTCAATATCATCTTCTGTCTCCGACGTTTCCGCTAGCTCGAACCAGG
	3R		GGATAACAATTTCACACAGGAAACAGCTGCGTTGCGAGTAGAACTTGC
**hph**	Fw	hygromycin B	GTCGGAGACAGAAGATGATATTGAAGGAGC
	Rv		GTTGGAGATTTCAGTAACGTTAAGTGGAT
